# Revisiting the dataflow principle for chemical information processing

**DOI:** 10.1186/1758-2946-4-S1-P15

**Published:** 2012-05-01

**Authors:** Wolf D Ihlenfeldt

**Affiliations:** 1 Xemistry GmbH, Königstein, Germany

## 

Dataflow systems, such as Pipeline Pilot or KNIME have become important mainstream tools for data processing in chemistry. These established systems are all implemented relying on a data model emphasizing a strict row/column-centric data table view which does not facilitate interaction with individual chemistry objects, or non-uniform data contents.

Resuming our pioneering work which resulted in the implementation of the first dataflow system for chemistry [1], we present in this contribution a different, object-centric approach for the design of re-usable chemical information processing sequences. Our system is based on the metaphor of a factory floor, instead of opaque pipelines. Individual machining stations perform configurable processing steps on objects such as structures, reactions, datasets or tables. Objects are transported between these - or temporarily set on the factory floor for storage or inspection. The combination of this general concept with the extensive scripting functionality of the Cactvs Chemoinformatics toolkit results in a system with capabilities notably different and more flexible than standard pipelining systems.
